# Endothelial cell-derived extracellular vesicles impair the angiogenic response of coronary artery endothelial cells

**DOI:** 10.3389/fcvm.2022.923081

**Published:** 2022-07-19

**Authors:** Nigeste Carter, Allison H. Mathiesen, Noel Miller, Michael Brown, Ruben M. L. Colunga Biancatelli, John D. Catravas, Anca D. Dobrian

**Affiliations:** ^1^Department of Physiological Science, Eastern Virginia Medical School, Norfolk, VA, United States; ^2^Frank Reidy Research Center for Bioelectrics, Old Dominion University, Norfolk, VA, United States; ^3^School of Medical Diagnostic and Translational Sciences, College of Health Sciences, Old Dominion University, Norfolk, VA, United States

**Keywords:** exosomes, barrier function, endothelial cells, proliferation, adipose tissue, wound response, miRNA

## Abstract

Cardiovascular disease (CVD) is the most prominent cause of death of adults in the United States with coronary artery disease being the most common type of CVD. Following a myocardial event, the coronary endothelium plays an important role in the recovery of the ischemic myocardium. Specifically, endothelial cells (EC) must be able to elicit a robust angiogenic response necessary for tissue revascularization and repair. However, local or distant cues may prevent effective revascularization. Extracellular vesicles (EV) are produced by all cells and endothelium is a rich source of EVs that have access to the main circulation thereby potentially impacting local and distant tissue function. Systemic inflammation associated with conditions such as obesity as well as the acute inflammatory response elicited by a cardiac event can significantly increase the EV release by endothelium and alter their miRNA, protein or lipid cargo. Our laboratory has previously shown that EVs released by adipose tissue endothelial cells exposed to chronic inflammation have angiostatic effects on naïve adipose tissue EC *in vitro*. Whether the observed effect is specific to EVs from adipose tissue endothelium or is a more general feature of the endothelial EVs exposed to pro-inflammatory cues is currently unclear. The objective of this study was to investigate the angiostatic effects of EVs produced by EC from the coronary artery and adipose microvasculature exposed to pro-inflammatory cytokines (PIC) on naïve coronary artery EC. We have found that EVs from both EC sources have angiostatic effects on the coronary endothelium. EVs produced by cells in a pro-inflammatory environment reduced proliferation and barrier function of EC without impacting cellular senescence. Some of these functional effects could be attributed to the miRNA cargo of EVs. Several miRNAs such as miR-451, let-7, or miR-23a impact on multiple pathways responsible for proliferation, cellular permeability and angiogenesis. Collectively, our data suggests that EVs may compete with pro-angiogenic cues in the ischemic myocardium therefore slowing down the repair response. Acute treatments with inhibitors that prevent endogenous EV release immediately after an ischemic event may contribute to better efficacy of therapeutic approaches using functionalized exogenous EVs or other pro-angiogenic approaches.

## Introduction

Cardiovascular disease (CVD) is the most prominent cause of death of adults in the United States, killing approximately 655,000 Americans each year (1). Coronary artery disease, the most common type of CVD, can lead to myocardial infarction, defined by myocardial cell death due to prolonged ischemia (2). The extent of ischemia-induced damage to the myocardium greatly depends on the duration of ischemia and efficient tissue repair. Thus, a timely and robust repair response is imperative for maintaining normal organ function. Specifically, successful reperfusion paired with *de novo* formation of blood vessels after a myocardial infarction is necessary not only to repair the ischemic myocardium but also to prevent heart failure by promoting left ventricular remodeling (3). The coronary endothelium plays an important role in this repair response following an ischemic event. Under physiological conditions, ECs are quiescent and thus contribute to the maintenance of blood flow and barrier function while counteracting thrombosis and vascular inflammation (4). However, following a myocardial event, ECs become activated, increasing vascular permeability and angiogenesis. Following an initial inflammatory response, a reparative phase ensues resulting in attenuation of inflammation, fibroblast proliferation, and neovascularization via angiogenesis (3, 5). Angiogenesis is a tightly regulated process controlled via multiple cues such as hypoxia, vascular endothelial growth factor (VEGFA), nitric oxide (NO), or other local mediators (6). When exposed to angiogenic signals, quiescent ECs become activated, loosening their tight junctions, and thus allowing extravasation of plasma proteins forming a provisional matrix. This matrix allows specialized ECs, termed tip cells, to migrate initiating the formation of a new sprout. Furthermore, neighboring EC, stalk cells, proliferate to further elongate the sprout. Individual elongated sprouts will then coalesce, forming a lumen and new vascular networks (6). Therefore, cell migration, proliferation and increased vascular permeability have a concerted contribution to successful angiogenic responses. Despite this well-regulated process, several paracrine or endocrine factors may impact on EC angiogenic response. One emerging player in the modulation of angiogenesis dependent or independent of inflammatory mediators are extracellular vesicles (EVs) (7, 8). EVs are a heterogeneous population of lipid bilayer encapsulated cargo that are secreted by virtually all cell types under both basal and pathological conditions (9). These vesicles contain cargo including lipids, proteins, microRNAs and other non-coding RNAs which can contribute to the modulation of biological function in recipient cells. Specifically, ECs are major contributors to the EV pool in the systemic circulation and the heterogeneity inherent to endothelium is reflected by their EVs released in circulation (9). EC-derived EVs can deliver cargo such as proteins, mRNA, and miRNA that may modulate the proliferation, migration, and angiogenic capabilities of recipient endothelial cells (8, 10, 11). Also, EVs from mesenchymal stem cells released under hypoxic conditions were shown to support the angiogenic process following myocardial ischemia (12). Recent studies also emphasized the beneficial role of EVs released after remote ischemic conditioning for myocardial protection and angiogenesis (13). Despite evidence for the beneficial roles of EVs from various cellular sources on cardiac remodeling and repair in pre-clinical models, these were not yet translated into effective therapies for patients with MI. A thorough understanding of the complex interplay between EVs from various sources and the local microenvironment in the heart are therefore of prime importance to ensure efficacy and safety of promising therapeutic approaches using EVs. Some evidence shows that EVs can also reduce angiogenesis *in vitro* and *in vivo* (8). Direct and indirect evidence indicates that EVs from adipose and other distant tissues can effectively reach the cardiac coronary circulation and induce functional effects in the endothelium and cardiomyocytes (13–15). Published data from our laboratory showed that EVs released by human adipose tissue ECs exposed to chronic inflammation have angiostatic effects on naïve, proliferating ECs from adipose tissue *in vitro* (16). This paracrine effect can reduce vascular remodeling in an expanding adipose tissue resulting in local inflammation and insulin resistance. Angiostatic EVs could also impact on coronary circulation and prevent effective post-ischemic re-vascularization. Also, it is currently unclear whether this effect is specific to EVs from adipose tissue endothelium or is also a feature of coronary EC-EVs exposed to pro-inflammatory cues. The objective of this study was to investigate the angiogenic effects of EVs produced by adipose microvasculature (heterologous) or coronary artery (autologous) ECs exposed to pro-inflammatory cytokines (PIC) on naïve coronary artery ECs. Specifically, we measured EVs effects on functional aspects that support angiogenesis, such as barrier function, migration and proliferation. We also determined the potential functional miRNA cargo in the EVs that may provide mechanistic hypotheses to the observed functional effects.

## Materials and methods

### Endothelial cell culture

Human coronary artery endothelial cells (HCAEC) were cultured using either complete EGM-2MV Bullet Kit media (Lonza, catalog no. CC-3202) or MesoEndo Cell Growth Medium (Cell Application, catalog no. 212-500) in a 37°C, 5% CO_2_ incubator. Human adipose microvascular endothelial cells (HAMVEC) were cultured on fibronectin-coated plates using complete Endothelial Cell Media (ScienCell catalog no. 1001) in a 37°C, 5% CO_2_ incubator. Experiments were conducted between passages 5–8 for both cell types. Endothelial cells were treated with pro-inflammatory cytokines, 5 ng/mL of TGF-β, IFN-γ, and TNF-α (designated as PIC) for 6 days, with a media change and addition of fresh cytokines after 3 days, as previously described (17). All culture conditions were performed in the above respective media. The same media was used for all the functional assays. Please note that media contained 2% FBS and growth factors including VEGF, for optimal cell growth and as recommended by manufacturer.

### Extracellular vesicles isolation and characterization

The cell culture media from untreated or PIC-treated HCAEC and HAMVEC seeded in T-150 flasks was collected after 6 days of treatment. The conditioned media was centrifuged at 500 × *g* for 10 min to remove dead cells, and the supernatant was collected and further centrifuged at 9,100 × *g* for 40 min to remove small cellular debris and larger particulates. The supernatant was retained, and vesicles were isolated by subsequent ultracentrifugation at 100,000 × *g* for 90 min. The supernatant was removed, and pelleted vesicles were resuspended in PBS and washed using an additional ultracentrifugation at 100,000 × *g* for 90 min. The supernatant was discarded, and the pellet containing purified EVs was resuspended in 500 μL of PBS. To determine concentration and size distribution, EVs were diluted 1:100 in PBS and nanoparticle tracking analysis was performed using NanoSight 300 (Camera Level: 13–15, Screen Gain: 1, Capture Number: 3, Capture Time Length: 10-s, Temperature: 25°C). Expression of extracellular vesicle tetraspanin expression was determined using the ExoView R100 platform (NanoView Biosciences, Brighton, MA, United States).

### Extracellular vesicles labeling

The lipophilic dye Vybrant DiD (Thermo Fisher Scientific, catalog no. V22887) was used to label autologous or adipose endothelial cell-derived EVs. Vybrant DiD dye was diluted 1:200 with PBS containing EVs and incubated for 20 min at 37°C. Unbound dye in the vesicle preparation was removed by ultracentrifugation at 100,000 × *g* for 90 min. The labeled pellet was then washed and resuspended in filtered PBS and spun again at 100,000 × *g*. The final labeled EV pellet was kept in filtered PBS, at 4°C for 24 h.

### Uptake of extracellular vesicles using imaging flow cytometry

Human coronary artery endothelial cell (100,000 cells) were seeded onto a six-well plate and grown until approximately 70% confluency. Cells were then incubated for 24 h with 50,000 EVs/cell of DiD-labeled EV-C or EV-PIC derived from autologous or heterologous ECs. The media was removed, and wells were washed with PBS. Cells were then treated with trypsin, pipetted through cell strainers into Eppendorf tubes, and pelleted at 220 × *g* for 5 min. The supernatant was discarded, and cell pellets were resuspended in 1 mL of PEB Buffer (1X PBS, 0.5% BSA, 2 mM EDTA, pH 7.4) followed by centrifugation at 220 × *g* for 5 min. Cells were then fixed on ice in 2% formaldehyde/PBS for 20 min and protected from light. After fixation, the cells were pelleted at 220 × *g* for 5 min, resuspended in 1:1000 DAPI solution, and incubated for 5 min. Cells were then pelleted at 220 × *g* for 5 min, supernatant was removed, and cells were resuspended in 30–40 μL 2% Fetal Bovine Serum/PBS (FBS, ScienCell cat no. #050). Cells were analyzed on an AMNIS ImageStream Mark II instrument and acquisition, and analyses were completed using Ideas 6.2 software. Specifically, the Ideas internalization wizard was utilized to determine the percentage of cells in each population that internalized EVs. To do so, the wizard uses a mask feature to calculate the intensity of DiD-stained EVs found within the masks placed over the brightfield image cells.

### *In vitro* tube formation

Human coronary artery endothelial cell were grown on six-well plates until approximately 70% confluency was reached. Cells were then incubated with either autologous or adipose EC-derived EV-C or EV-PIC for 24 h. Prior to seeding on Matrigel, cells were >90% confluent, approaching quiescence. Growth factor reduced Matrigel (Corning, catalog no. 356231) was thawed overnight on ice at 4°C. The Matrigel was then diluted 1:1 with EGM-2MV Bullet Kit Media or MesoEndo Cell Growth Medium, and 150 μL of the diluted Matrigel was aliquoted into wells of a 48-well plate. Plates were then incubated at 37°C, 5% CO2 for 35 min to allow the Matrigel to settle. Following EV treatment, HCAEC were incubated with 1 g/mL calcein AM in growth media for 30 min at 37°C, 5% CO2, and excess dye was removed by 2 × PBS washes. Following incubation with calcein AM, cells were treated with trypsin, and each well was seeded with 35,000 HCAEC and allowed to grow in a 37°C, 5% CO_2_ incubator for 6 h. Cells were then imaged using an Olympus 72 fluorescent microscope using 40× magnification. Average mesh formation was assessed using ImageJ Angiogenesis Analyzer software.

### Endothelial cell proliferation

50,000 HCAEC were seeded into four-well chambered slides and grown until approximately 70% confluent. Cells were incubated with EV-C or EV-PIC from autologous or adipose ECs for 24 h. Following vesicle treatment, cells were incubated with 10 μM BrdU (Abcam, catalog no. ab142567) in growth media for 16–18 h. BrdU labeling solution was removed from the wells, and cells were washed 3× with PBS. The cells were then fixed with 2% paraformaldehyde/PBS at room temperature for 30 min. Wells were washed 3× with PBS, and permeabilized with 0.1% Triton X-100 for 30 min. Wells were then washed 3× with PBS, and cells were hydrolyzed with 2M hydrochloric acid for 20 min at 37°C. Hydrochloric acid was removed and sodium tetraborate was added for 30 min at room temperature. Wells were washed 3× with PBS, and cells were blocked with 10% Normal Goat Serum (NGS; Vector Labs, cat#: S-1000) for 1 h. After additional three washes with PBS, the cells were incubated with anti-BrdU Alexa Fluor 488 antibody (Santa Cruz Biotechnology, catalog no. SC-32323 AF488) and 5 μg/mL DAPI prepared in 10% NGS. Wells were washed 3× with PBS, and coverslipped with Fluoromount-G. Three images/well in duplicate wells were captured and further analyzed. Representative images were taken at 200× with an Olympus BX50 fluorescence microscope.

### Endothelial barrier function and wounding

Human coronary artery endothelial cells (HCAEC) were seeded on 96-well gold electrode arrays (96W10idf), and endothelial barrier integrity was assessed by Electric Cell-Substrate Impedance Sensing (ECIS) technique, using an ECIS model 1600R ζθ instrument (Applied Biophysics). Approximately 50,000–75,000 cells were seeded in each well, let grow overnight and experiments were conducted when a stable resistance was achieved above 800 Ω, which is indicative of a confluent monolayer (18). Cells were then exposed to autologous or heterologous EVs isolated from conditioned culture media of human coronary EC (HCA) or human adipose tissue EC (HAM), cultured in presence or absence of pro-inflammatory cytokines (EV-C or EV-PIC). For wounding assays, cells were pre-treated with different types of EVs and then pulsed with 3000 μAmps for 30 s. A number of 3–5 EV preparations for each experimental condition were used. Resistance values were collected and normalized to each well’s value at *t* = 0. Data are represented as mean values (±SEM). Results were considered significant when *p* < 0.05 with two-way ANOVA and Bonferroni’s *post hoc* test. Also, time-dependent resistivity was plotted for 3 discrete time points and the area under the curve (AUC) of the normalized TER values was computed and compared using the non-parametric Mann–Whitney test.

### Nanostring miRNA analysis

Human coronary artery endothelial cell and HAMVEC were grown in T-150 flasks until they reached approximately 70% confluency. Cells were then treated with control or PIC media. Following 6 days of treatment, conditioned media was harvested, and EVs were isolated, as previously described (17). Total RNA from vesicles was extracted using Norgen Single Cell RNA Purification Kit (Biotek Corporation, catalog no. 51800). Extracellular vesicle RNA were analyzed for expression of 800 miRNAs using Human v3 miRNA panel (Nanostring Technologies, Seattle, WA, United States). Nanostring nCounter software was used to normalize the results. Experimentally validated miRNA targets and downstream pathways were identified using Qiagen ingenuity pathway analysis (IPA) miRNA target filter analysis tool in conjunction with PubMed literature review.

### Senescence assay

100,000 cells were seeded into six-well plates and allowed to grow to 50–60% confluency, and cells were treated EV-C or EV-PIC overnight. Cells were stained for β-galactosidase according to the Senescence β-Galactosidase Staining Kit (Cell Signaling Technology, cat#:9860). Briefly, the endothelial growth media was removed from the plate and washed once with PBS. Wells were incubated with 1X fixative for 15-min, and the plate was washed twice with PBS. 1.0 mL of β-galactosidase staining solution (pH 6) was added to each well, and the plate was covered and wrapped in parafilm. The plate was stored for 18-h in a non-CO_2_ incubator. Cells were then washed with PBS and stained with DAPI (1:1000 for 5 min) and washed once more with PBS. β-galactosidase staining was recorded using bright-field microscopy and DAPI staining was recorded using fluorescent microscopy. Both images were taken using an Olympus IX 73 microscope. Senescence was reported as the percent beta-galactosidase positive cells relative to total number of cells as detected by DAPI staining.

### Statistical analysis

Statistical analysis was performed using GraphPad Prism Software v7.03 (GraphPad Software). Data is expressed as the mean ± standard deviation. Normality assumptions were verified using Shapiro–Wilk tests. If data passed assumptions, student’s *t*-test was performed for comparisons of two groups, and ANOVA was performed for comparisons of three or more groups. A Tukey’s HSD test was performed for *post hoc* analysis of groups when utilizing ANOVA. If data failed either assumption, non-parametric tests, such as Wilcoxon Rank, Kruskal–Wallis, and Mann–Whitney tests were conducted to determine significance. The null hypothesis was rejected for *p*-value < 0.05.

## Results

### Endothelial cells release heterogeneous extracellular vesicles that are internalized by human coronary artery endothelial cell

**FIGURE 1:**
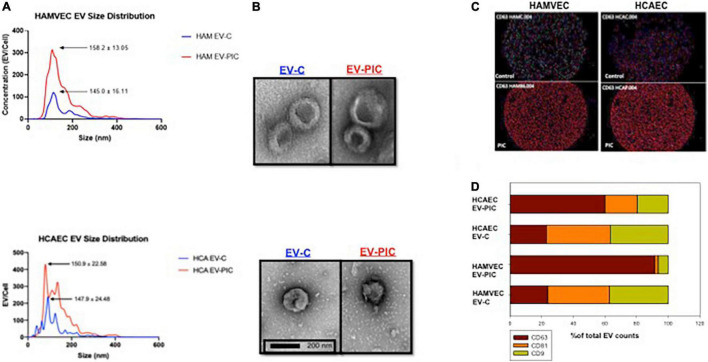
Production and characterization of autologous (human coronary artery – HCAEC) and heterologous (human adipose tissue – HAMVEC) endothelial cell EVs in response to pro-inflammatory cytokines (PIC) treatment. **(A)** Nanoparticle tracker analysis found similar size distribution of EVs isolated from untreated cells (EV-C) and from PIC-treated cells (EV-PIC) for both autologous and heterologous EVs. Please note that PIC treatment resulted in a 2–3-fold increase in EV numbers/cell compared to untreated controls for both populations of EVs. **(B)** Representative micrographs of negative staining electron microscopy for EV-C and EV-PIC from HCAEC and HAMVEC sources. EM confirms the EV size range calculated by nanoparticle tracking analysis in both preparations and illustrates the typical donut-shaped morphology of the EVs from both cellular sources. **(C)** Fluorescent images of ExoView chips of EVs from HAMVEC (left) and HCAEC (right) showing expression of the EV marker CD63. Please note that EVs from both cellular sources that were treated with PIC showed a strong increase in CD63 expression. **(D)** Quantification of relative fluorescence for the tetraspanins CD63, CD9, and CD81 using the ExoView technology shows the different relative expression of each EV population. Similar tetraspanin relative expression was found for EVs from control HAMVEC and HCAEC and increased expression of CD63 was found on EVs from both type of endothelial cells treated with PIC.

Coronary artery and adipose microvascular ECs were treated for 6 days with a combination of TNF-α, IFN-γ, and TGF-β (PIC) (5 ng/mL each). In our previous study, we used this combination as it was shown to be increased in adipose tissue of obese humans (19, 20). Furthermore, this combination of cytokines has been shown to also be increased in CVD and ischemic injuries, and was used at similar concentrations in various *in vitro* studies involving coronary artery ECs (21–24). EVs were collected from conditioned media of control (EV-C) or PIC (EV-PIC) treated ECs and separated using differential ultracentrifugation. We found that both coronary artery and adipose microvascular ECs treated with PIC release ∼2–3-fold more EVs compared to untreated, control ECs (Figure 1A). EVs from control ECs have similar size distributions with an average peak around 140 nm, while ECs treated with PIC are on average slightly larger, with an average peak between 150 and 158 nm (Figure 1A). EV size was confirmed by negative staining electron microscopy and their morphology showed the typical cup-shaped feature (Figure 1B).

We further confirmed the presence of the typical tetraspanin EV markers CD63, CD81, and CD9 using the ExoView R100 platform and the proprietary antibody array chips (Figures 1C,D). Specifically, EVs were captured on chips containing CD63 antibodies, incubated with a fluorescent antibody cocktail and imaged. While all EV subtypes displayed various levels of expression of the three small EV markers, there was a clear shift in their tetraspanin profile following PIC treatment (Figure 1C). In the autologous EV population from PIC conditioned media, 62% of the EVs expressed CD63 compared to only 25% of the control EV population. The heterologous (adipose-derived) EV population from EC treated with PIC showed CD63 positivity for ∼95% of the EVs compared to 25% for the EVs from control cells (Figure 1D). These results show that the tetraspanin profile of EC-derived EVs may change in response to proinflammatory cytokines, which may play a role in EV uptake and by target cells and possibly their functional outcome.

**FIGURE 2:**
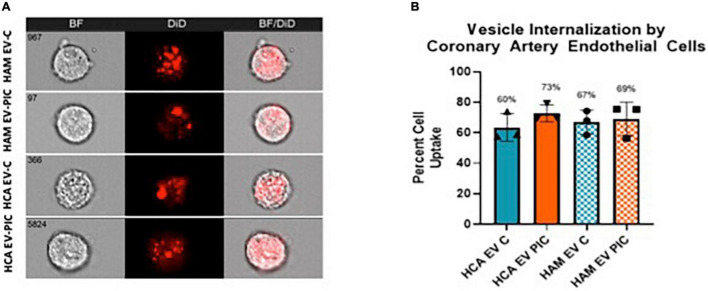
EV uptake by human coronary endothelial cells (HCAEC) is independent of EV source. **(A)** HCAEC were incubated with DiD labeled HCAEC or HAMVEC EV C or EV PIC for 24 h prior to imaging flow cytometry. The number of cells that showed a detectable positive signal was determined using the IDEAS software internalization wizard. **(B)** Uptake was calculated as percentage of cells that were positive for the DiD fluorescence following the background subtraction of control cells incubated with EV-free dye. Uptake was similar for all the EV populations (*n* = 3 individual preparations). Non-parametric ANOVA found a *p*-value = 0.649. HAMVEC, human adipose tissue microvascular endothelial cells; EV C, extracellular vesicles isolated from control cells; EV PIC, extracellular vesicles isolated from PIC-treated cells.

Next, we compared the cellular internalization of autologous (HCA EV-C and HCA EV-PIC) and heterologous (HAM EV-C and HAM EV-PIC) EVs by HCAEC at 70–80% confluency, which mimics a low proliferative state for ECs. The internalization of fluorescently labeled EVs was determined by imaging flow cytometry after incubation of coronary EC with 50,000 EVs/cell for 24 h (Figure 2). Titration experiments were performed to determine the kinetics of internalization and found a saturable effect reached for 50,000 EVs/cell. Comparison of percent cells that show a positive signal for internalization for different EV populations showed that there were no differences in the numbers of cells that internalized the EVs (Figure 2B). This result indicates that the cells have a similar internalization capacity regardless whether the EVs originate from autologous or heterologous sources and, the pro-inflammatory environment has no significant effects on EV internalization.

### Endothelial cell-derived vesicles have angiostatic effects on human coronary artery endothelial cell and reduced their proliferation

**FIGURE 3:**
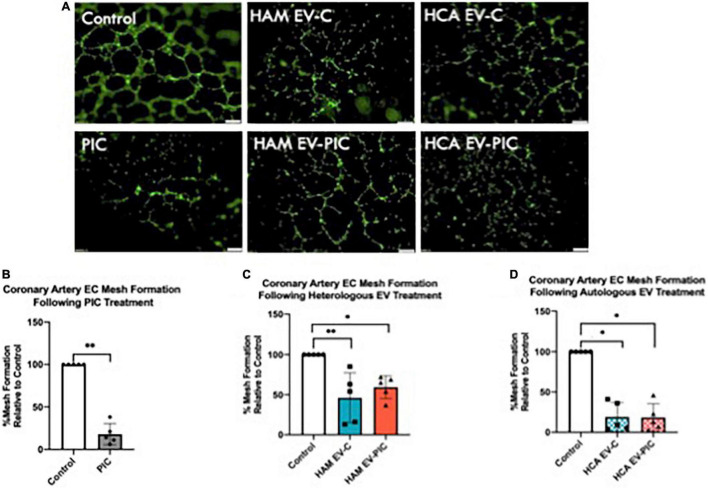
Extracellular vesicles (EV) from control and pro-inflammatory cytokines (PIC) treated endothelial cells reduce *in vitro* tube formation. **(A)** Coronary artery endothelial cells were incubated for 24 h with EV-free media containing VEGF, PIC + VEGF media, or EV from control or PIC-treated HAMVEC (HAM EV-C or HAM EV-PIC) or HCAEC (HCA EV-C or HCA EV-PIC) in presence of VEGF. After treatment, cells were labeled with calcein, seeded in growth factor reduced matrigel coated plates and incubated for 6 h before imaging. Representative micrographs show reduced formation of complete mesh when treated with EVs from all cellular sources; Magnification 40× (Scale bar = 200 μm). **(B–D)** Quantitative analysis of tube formation using the ImageJ software. Data is expressed as percent of mesh reduction relative to control (set at 100%) (*n* = 5). Statistical analysis was done using the Mann–Whitney non-parametric test. The null hypothesis was rejected for a *p* < 0.05. *p < 0.05; **p < 0.01.

To determine the angiogenic potential of the EVs on coronary endothelium, we performed *in vitro* tube formation assays in the presence of VEGFA. Coronary cells require VEGF to proliferate *in vitro* and absence of VEGF from media completely prevents tube formation. Following 24-h exposure to 50,000 EVs/cell from either autologous or heterologous sources in absence or presence of PIC, mesh formation was measured and compared with control coronary cells without EV treatment (Figure 3). Mesh formation is a measure of coordinated development of tips and branches, and it illustrates an angiogenic response leading to a primitive network of EC that forms vascular lumens. We found that in contrast with control cells, HCAEC treated with EVs show varying levels of incompleteness of mesh formation that suggests an imbalance between sprouting, elongation and branching of the network (Figure 3A). Heterologous EVs (HAM-EV), regardless of PIC exposure of the parental cells, led to a significant reduction by ∼50% of the mesh compared to control, untreated HCAECs (Figure 3C). Autologous EVs (HCA EV) had a more dramatic effect leading to almost complete abolition of the mesh structure and, the effect was equally potent regardless exposure of parental cells to PIC (∼75% reduction in mesh formation compared to untreated control) (Figure 3D). This effect was comparable with the one resulting from direct addition to the media of the PIC, in the absence of EVs (Figure 3B). Since EVs were added to the HCAEC prior to their seeding in matrigel it is unlikely that the EVs directly interfered with VEGF binding to its receptor. However, other mechanisms that involve post-receptor signaling may have direct or indirect impact on functions such as cell proliferation or migration. Therefore, this data led us to assess other functional parameters required for angiogenesis, such as proliferation, barrier function, and cell migration to identify potential mechanisms underlying the angiostatic effect.

**FIGURE 4:**
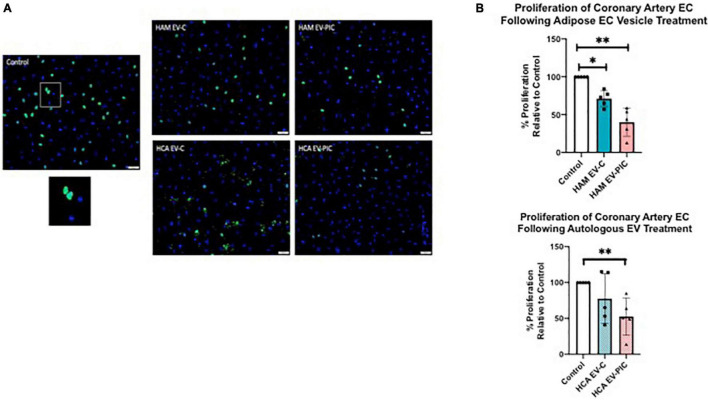
EVs isolated from both PIC treated HAMVEC (heterologous) and HCAEC (autologous) conditioned media decrease HCAEC proliferation. **(A)** Representative micrograph of control cells. Cell nuclei are stained with DAPI (blue). Nuclei of the proliferating cells are labeled by a FITC- BrdU antibody (green). Overlapping images of the same field show both proliferating cells (light blue nuclei) and non-proliferating cells (dark blue, DAPI only). Magnification is 200×. **(B)** Quantitative analysis of HCAEC proliferation expressed as percent cells showing BrdU incorporation of total cells. Data is expressed as percentage of proliferation for EV-treated cells relative to untreated controls (set at 100%). Statistical analysis was performed using the Mann–Whitney non-parametric test (**p = 0.0059; *p = 0.0459).

Since mesh formation was significantly reduced by EVs, we measured the impact of the latter on the proliferative capacity of coronary endothelial cell measured by BrdU incorporation (Figure 4). HCAEC at 60–70% confluency were treated with 50,000 EVs/cell for 24 h prior to measurement of proliferation. Heterologous EVs (HAM EV) significantly reduced proliferation compared to untreated controls, on average by 28 and 63% in the absence or presence of PIC treatment of the parent cells, respectively (Figures 4A,B). Autologous EVs produced by cells treated with PIC (HCA EV-PIC) significantly decreased EC proliferation by ∼50% compared to controls, while HCA EV-C produced by cells in absence of inflammatory cytokines had no significant effect on coronary EC proliferation, although they displayed a trend toward decreased proliferation (Figures 4A,B). Based on this data, it appears that heterologous EVs may lead to a decreased mesh formation by HCAEC, at least in part, due to slowing down the rate of EC proliferation. This scenario is also supported by data from HCAEC exposure to autologous EVs from PIC treated cells, but not from cells in absence of an inflammation. Since reduced rates of proliferation may be due to an increase in the number of senescent cells within the EC population, we also measured the effect of EVs on HCAEC senescence. We have found that, regardless of their cellular source and presence of PIC, EV did not have an effect on the percent of senescent cells, compared to untreated controls, as measured by a b-galactosidase assay (Supplementary Figure 1).

### Endothelial cell-derived extracellular vesicles modulated human coronary artery endothelial cell response to wounding and barrier function

**FIGURE 5:**
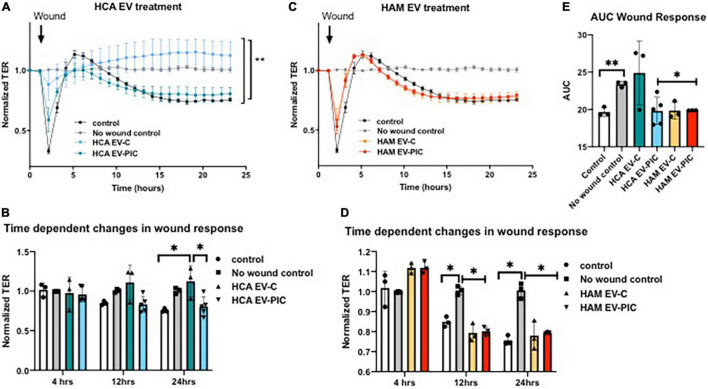
Pretreatment with autologous and heterologous EVs modulates HCAEC response to wounding. Following 24 h of treatment with different EV preparations, the cell monolayer was disrupted by the application of an electric pulse of 3000 μAmps for 30 s. A number of 3–5 EV preparations were used in triplicates for each experimental condition. **(A,B)** Time-dependent recording of normalized TER indicates full recovery of coronary cells treated with autologous EVs (HCA EV-C) 24 h after wounding. Control cells with no EV treatment and autologous EVs produced by PIC treated cells (HCA EV-PIC) failed to fully recover 24 h post-wounding. **(C,D)** All coronary cells following wounding maintained significantly lower barrier function at 12 h and up to 24 h post-wounding compared to control cells with no wound. **(E)** Area under the curve (AUC) was calculated using the normalized TER data from the time of wounding and over the following 24 h. Resistance was recorded continuously at 600 s intervals and normalized to *t* = 0. Means ± SEM; *p < 0.05; ^**^p < 0.01 with 1- or 2-way ANOVA and Bonferroni’s *post hoc* test **(A)** and Mann–Whitney non-parametric test **(B,D,E)**.

The reduced potential of HCAEC exposed to EVs to form a tubular network *in vitro* can be caused by impaired migration of EC. It is recognized that the ability of tip cells to migrate toward VEGF gradients is key for the initiation of angiogenic sprouts *in vivo*. We used an indirect measure of migratory function of HCAEC based on their capability to restore the monolayer integrity in response to wounding. HCAEC were seeded on a 96 wells 10idf array and connected to ECIS 1600 R (zeta-theta) instrumentation until they reached confluency. Autologous and heterologous EVs, obtained from unstimulated or stimulated (PIC-treated) cells, were added to HCAEC at a concentration of 50,000 EVs/cell and after 48 h an electric pulse of 3000 μAmps was applied for 30 s in 10 discrete areas of the monolayer. Recovery from wound was measured over 24 h (Figure 5). Pre-treatment with autologous EVs from control unstimulated cells (HAM EV-C) led to a rapid and full recovery of the cell monolayer post-wounding (Figures 5A,B). At the end of the 24-h measurements the cells pre-treated with EVs from cytokine stimulated HCAEC (HCA EV-PIC) had significantly lower normalized transendothelial resistance than the HAM EV-C treated cells, indicating a significant reduction in wound recovery due to reduced migration (Figures 5A,B). Interestingly, control cells in the absence of EV treatment did not fully recover from wounding, compared to a no wound control. This suggests, that autologous EVs may support endothelial repair following injury. Pre-treatment with heterologous EVs (HAM EV-C and HAM EV-PIC) did not change the wound recovery compared to control non-treated ECs. None of the cell monolayers post-wounding fully restored their integrity (Figures 5C,D). The integrated wound response over 24 h, expressed as the area under curve (AUC) of the normalized TER further indicates the unique restorative response of HCAEC pre-treated with autologous EVs from control, unstimulated cells (Figure 5E). However, the angiostatic effect of EVs on the coronary endothelium cannot be directly attributed to differences in EC migration, at least under the methodological approaches used in these experiments.

**FIGURE 6:**
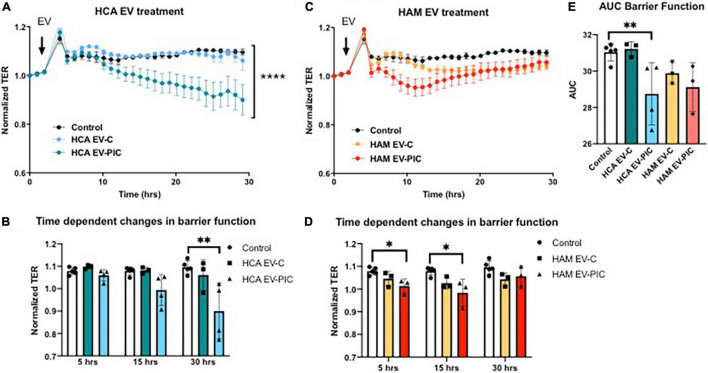
Effect of autologous and heterologous EVs on human coronary artery endothelium barrier function. HCAEC were seeded on 96W10idf array until confluent, i.e., until stable resistance was achieved (>800 Ω). Three to five EC preparations were measured in triplicates for each experimental condition. **(A,B)** Treatment with autologous EVs from HCAEC exposed to PIC (HCA EV-PIC) significantly reduced barrier function compared to untreated controls. This effect is time-dependent and becomes significant after 24 h. Autologous EVs from untreated cells (HCA EV-C) did not have a significant effect on EC barrier function and the integrity of the cell monolayer was maintained throughout the experiment. **(C,D)** Heterologous EVs produced by cells in the presence of pro-inflammatory cytokines (HAM EV-PIC) exhibit early and transient reduction in barrier function at 5 and 15 h. EVs collected from adipose microvascular cells in the absence of cytokine treatment (HAM EV-C) did not have a significant effect on barrier function. **(E)** Area under the curve (AUC) was computed using the normalized TER values over a 30-h interval. Resistance was recorded continuously at 600 s intervals and normalized to *t* = 0. Means ± SEM; *p < 0.05; ^**^p < 0.01; ****p < 0.0001 with two-way ANOVA for repeated measures and Bonferroni’s *post hoc* test **(A,C)**; Mann–Whitney non-parametric test **(B,D,E)**.

Initiation of angiogenesis is associated with an increase in EC monolayer permeability and the loosening of endothelial junctions. Therefore, we measured endothelial barrier function in response to EV treatment. Under basal conditions, the vascular endothelium forms a semi-permeable barrier that controls the transport and passage of molecules. However, chronic inflammation and extracellular vesicles have both been shown to contribute to vascular endothelial barrier dysfunction (4, 25). Autologous EVs from PIC treated cells (HCA EV-PIC) provoked a decrease in barrier function, indicating increased permeability of the cell monolayer (Figures 6A,B). This effect became significant 20 h after EV addition and was maintained for the following 10 h (Figure 6B). The effect was unique to the autologous HCA EV-PIC, as EVs from control autologous cells (HCA EV-C) did not elicit any changes in barrier function over 30 h of EV exposure, compared to untreated controls (Figures 6A,B). Exposure to heterologous EVs from cells treated with PIC (HAM EV-PIC) caused a transient decrease in barrier function, starting as early as 5 h after exposure to EVs that recovered after 15 h. Heterologous EVs from untreated cells (HAM EV-C) did not significantly change the transendothelial resistance throughout the experiment (Figures 6C,D). The integrated normalized TER over 30 h, calculated as the AUC, showed a significant decrease in response to autologous EVs from PIC stimulated cells only, suggesting a sustained long-term response (Figure 6E). In addition to reduced proliferation, reduced barrier function may be an independent contributor to angiostatic effects of autologous EVs from PIC stimulated cells.

### miRNA cargo composition in control and pro-inflammatory cytokines-treated endothelial cell extracellular vesicles

**FIGURE 7:**
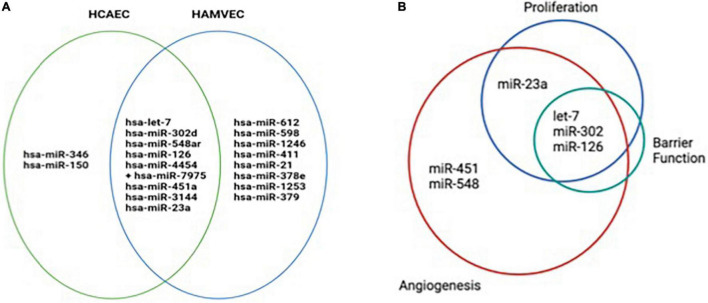
Venn diagrams showing miRNA cargo and predicted functional effects of EVs from human coronary artery cells (HCAEC) and human adipose tissue microvascular endothelial cells (HAMVEC). **(A)** HCAEC and HAMVEC EV C and EV PIC miRNA cargo was measured using the Nanostring miRNA array. Three different EV preparations were used for each experimental condition and only the miRNA that were detectable in all three EV preparations are shown. **(B)** Predicted gene targets for all the miRNA detected in the different EV populations were obtained using the IPA miRNA target filter tool and further sorted by the number of their target genes into functional pathways that included angiogenesis, proliferation, and barrier function. The greatest proportion of miRNA involvement was found to be in angiogenesis, followed by proliferation, with the fewest miRNA targets involving barrier function.

Many of the functional effects of EVs on recipient cells are attributed to their miRNA cargo. We employed an unbiased analysis of the miRNA cargo of EVs using an 800 miRNA panel of the Nanostring platform. A number of 18 miRNA were detectable in the EVs, with 8 miRNA shared by EVs produced by HCAEC and HAMCEV cells (Figure 7A). HCAEC had 2 unique miRNAs – miR-346 and miR-150, while HAMVEC had 8 unique miRNAs (Figure 7A). Since both autologous and heterologous EVs had angiostatic effects, IPA miRNA target filter tool was used to identify experimentally validated target genes of the shared miRNA, to identify functional miRNA cargo candidates (Table 1). Eight miRNA were identified in both HCAEC and HAMVEC EV C and EV PIC, let-7, miR-302, miR-548, miR-126, miR-23a, miR-451, miR-3144, and miR-4454 + 7975 (Figure 7A). These miRNAs were segregated based on their predicted functional impact on angiogenesis, proliferation and barrier function (Figure 7B). Six of the eight miRNA had predicted effects on angiogenesis (let-7, miR-302, miR-548, miR-126, miR-23a, and miR-451), four on proliferation (let-7, miR-302, miR-126, and miR-23a) and 3 on barrier function (let-7, miR-302, and miR-126). The latter had predicted effects on all the functional categories tested (Figure 7B).

**TABLE 1:** miRNA cargo shared by HCAEC and HAMVEC extracellular vesicles and their validated gene targets involved in angiogenesis, proliferation, and barrier function.

EV miRNA identified	Mean expression values (Counts)				Genes targeted			
	HCAEC		HAMVEC		Functional involvement			
	EV C	EV PIC	EV C	EV PIC	Angiogenesis		Proliferation	Barrier function
let-7	33.51	36.82	31.80	38.31	ACP1	LOXL2	ACP1	F2
					ADAMTS2	NRAS	ADAMTS2	RHOB
					ADGRG1	MTRR	COL1A1	
					BSG	MYC	COL1A2	
					CASP3	PDGFA	COL8A1	
					CCND1	PDGFB	DICER1	
					CDK6	PRDM1	F2	
					COL1A1	PTGS2	HMOX1	
					COL1A2	RAS	ITGB3	
					COL4A2	RHOB	LOXL2	
					COL5A1	RHOG	MYC	
					COL8A1	SPARC	NRAS	
					DICER1	TGFBR1	PDGFB	
					F2	TGFBR2	PTGS2	
					HMGA2	THBS1	RAS	
					HMOX1	TLR4	SPARC	
					ITGB3	VIM	TGFBR1	
					KRAS		TGFBR2	
					LAMC1		THBS1	
miR-302	210.44	379.95	58.50	55.58	ADAM9	LEFTY1	APP	VEGFA
					APP	NR4A2	CD44	
					CCND1	PRKACB	CDKN1A	
					CD44	RECK	DKK1	
					CDKN1A	RELA	NR4A2	
					DKK1	STK4	VEGFA	
					ESR1	VEGFA		
					LMNB1			
miR-23a	32.38	20.00	26.33	21.84	CXCL12	PLAU	CXCL12	
					HES1	PTEN	PLAU	
					IL6R	SMAD3	PTEN	
					LMNB1	SMAD4		
					MET	SMAD5		
					NOTCH1	TNFAIP3		
miR-126	33.51	31.05	45.03	28.45	CRKL	VCAM1	SPRED1	VEGFA
					IRS1	VEGFA	VEGFA	
					SPRED1			
miR-451	46.36	33.86	38.70	32.95	MIF			
miR-548	97.9	83.51	27.68	27.08	ERBB2			
miR-3144	122.91	224.37	27.01	28.38	No experimentally validated targets			
miR-4454 + miR-7975	30.58	31.05	35.76	26.80	No experimentally validated targets			

HCAEC and HAMVEC EV miRNA target genes were identified using the IPA target filter tool and sorted by involvement in angiogenesis, proliferation, and barrier function.

## Discussion

Endothelium is a major contributor to the maintenance of vascular homeostasis and a hub that coordinates responses to injury and repair in various vascular pathologies through inherent plasticity (9, 26, 27). Coronary endothelium is of prime importance for the maintenance of adequate vascular perfusion of the myocardium. Under- or over-perfusion may both have pathological effects due to inadequate levels of oxygen and nutrients. Importantly, partial or total occlusion of coronaries leads to significant ischemic damage to the heart that can result in pathologic remodeling or death. Timely re-perfusion via collateral re-vascularization and angiogenesis are key to restore cardiac function and physiologic remodeling of the myocardium. Current therapeutic approaches have limited success and are particularly challenging in aging populations and in individuals with co-morbidities such as obesity and type 2 diabetes. A promising contemporary approach involves stem cell therapy as a mean to repair the myocardium. However, cellular therapies that involve heterologous cells are challenging due to immune rejection and autologous cells may have limited therapeutic benefits due to reduced potency in elderly individuals or patients with obesity, or existing cardio-metabolic pathologies. Extracellular vesicles produced by stem cells are a more appealing alternative, as they can be generated in large amounts *in vitro* from stem cells of healthy individuals and are largely non-immunogenic (28). Some success has been reported in various pre-clinical models, but the variability in the response due to local and systemic microenvironment of the recipient remains a challenge (28). Therefore, amongst other variables, understanding better the complex interplay between endogenous EVs generated by different endothelia and the exogenous therapeutic EVs, is of prime importance to optimize such approaches. In this paper, we focused on understanding the effects of autologous coronary endothelial EVs and adipose tissue microvascular EVs, produced in physiologic or pro-inflammatory conditions, on coronary EC function. We selected these two human endothelial cell types due to relevance for the myocardial repair of their EVs as contributors from local microenvironment (coronary EC) as well as distant sites, such as adipose tissue (adipose microvascular EC); and representative of normal or obese conditions characterized by low grade inflammation (with or without PIC stimulation). We also built on previous data from our lab showing that EVs from adipose tissue EC in proinflammatory conditions are angiostatic for autologous naïve cells (17). Under physiologic conditions, ECs significantly contribute to the EVs in blood circulation. Several studies have shown that inflammation effectively increases EV release by ECs (29–32). In line with our previously published data, we found that endothelial cells from different vascular beds stimulated with proinflammatory cytokines released more EVs compared to control cells. By virtue of their higher numbers, EVs produced by ECs in an inflammatory environment may be more effective to transfer functional cargo by competing for uptake with EVs from other sources. However, we found that HCAEC internalized both autologous and heterologous EVs from control and PIC-stimulated cells at a similar rate, in a non-competing setup when ECs were treated with individual and equal numbers of each of the EVs populations. The exact mechanism of EV internalization by HCAEC is yet to be confirmed but likely involves receptor-mediated endocytosis (data not shown). We also found that autologous and heterologous EVs displayed the three tetraspanin exosomal markers, CD63, CD81, and CD9. Interestingly, when HAMVEC and HCAEC were exposed to pro-inflammatory cytokine treatment, there was a robust increase in CD63 expressing EVs (Figure 1). Although not extensively explored, some studies have shown that tetraspanins may interact with other transmembrane proteins to promote EV docking and selective tissue- and cell-dependent uptake (33, 34). ECs express transmembrane protein such as integrins and ICAMs, commonly found in tetraspanin-enriched microdomains (TEMs) that are used for immune cell adhesion (8, 35). Thus, such molecules could also be utilized by EVs for cellular targeting. Findings from animal models have shown that EVs from adipose tissue functionally impact the cardiomyocytes (15) and EVs from EC of peripheral beds subject to remote ischemia reperfusion have beneficial effects on cardiac pre-conditioning (13). Such studies provide an indirect proof that peripheral EVs can successfully reach the coronary endothelium. Conversely, EV produced by coronary endothelium can be potent modulators of immunity and inflammation post-MI and contribute to myocardial functional restoration via mobilization of neutrophils from spleen, thereby triggering an “autocrine” effect via an endocrine route (36). Based on this proven accessibility of EVs between coronary circulation and peripheral vascular beds, we speculate that our *in vitro* data should bear functional significance *in vivo*, in a pathologic context.

Effective angiogenesis is key to support an adequate repair response following a post ischemic myocardial event. In this study, we assessed the angiogenic potential of HCAEC following treatment with EVs derived from coronary or adipose endothelial cells stimulated with proinflammatory cytokines. We found that regardless of parental cell treatment and EC source, all EV preparations significantly impaired *in vitro* HCAEC tubule formation, a surrogate for angiogenic potential. Evidence for anti-angiogenic effects of EVs are by far less commonly reported compared to their pro-angiogenic effects (7). In the context of ischemic diseases, the effects of EVs on coronary ECs are reportedly pro-angiogenic and emphasized as promising therapeutic interventions. However, such EVs are primarily produced by stem or progenitor cells from either endogenous or exogenous sources, not by EC themselves (28). To our knowledge, the effect of autologous EVs in normal or pro-inflammatory conditions on coronary EC angiogenesis were not reported. Some of the mechanisms underlying inhibitory effects of EVs on angiogenesis include primarily mechanisms of uptake inhibition such as LDL receptor endocytosis or CD36-independent uptake or induction of oxidative stress [reviewed in Todorova., et al (7)]. Also, published data suggested that high concentrations of EC-derived EVs can induce apoptosis and impair angiogenesis in recipient ECs (37–39). Specifically, Ou et al. found that increased concentrations of EC-derived EV inhibited angiogenesis by inducing endothelial nitric oxide synthase (eNOS) dysfunction (39). Our data indicate that all the tested EV populations are taken up in an indiscriminate fashion by ∼65–70% of the HCAEC that approach confluence (Figure 2). Therefore, we focused our efforts to identify alternative mechanisms that can explain EVs inhibitory effects on HCAEC angiogenesis beyond their mechanism of uptake; specifically, we assessed proliferation, barrier function, and migratory response following EC monolayer wounding. Although all EV preparations reduced EC proliferation, only autologous EVs from PIC-stimulated cells reduced barrier function. Furthermore, control autologous EVs appeared to have aid in the reestablishment of the HCAEC monolayer following wound induction. Thus, EC-derived EVs impair several parameters required for mounting a sustainable angiogenic behavior. Notably, the most consistent and robust effect of EVs across all preparations was on EC proliferation. This included EVs from both PIC-treated and control HAMVEC and HCAEC cultures. Therefore, we propose that reduced proliferation may be necessary and sufficient for reduced angiogenesis, even in absence of a pro-inflammatory environment. As discussed below, some of the shared miRNA cargo support a uniform anti-proliferative effect of the EVs. In contrast with our findings, several studies have shown that EC-derived EVs promote angiogenesis and proliferation in recipient ECs (40–42). In one such study, EVs released by ECs contained β1 integrin and enzymatically active matrix metalloproteinases; these EVs were capable of inducing EC invasion and tubule formation *in vitro* (40). Furthermore, Lombardo et al. showed that EC stimulated with Interleukin-3 (IL-3) secreted EVs that promoted angiogenesis through the transfer of miR-126-3p and pSTAT5, and increased ERK1/2 activation in recipient EC (41). Nevertheless, further studies will be needed to fully understand the impact of EVs on endothelial cell proliferation and migration.

Extracellular vesicles are complexly involved in the regulation of endothelial barrier and increased permeability during infectious or inflammatory states (25). Indeed, chronic vascular diseases like atherosclerosis and diabetes display increased levels of plasma EVs originating from either platelet, endothelial cells or leukocytes, that promote microvascular leakage (43, 44). This has been further supported by the fact that exosome generation blockade by GW4869, and subsequent inhibition of EVs release, ameliorates systemic inflammation in mice (45). EVs isolated during inflammatory states showed to disrupt endothelial homeostasis and impair vasorelaxation and eNOS production (46).

We investigated if autologous and heterologous EVs, obtained in either unstimulated or inflammatory conditions, would influence the permeability of a monolayer of HCAEC and their response to wounding. Interestingly, autologous EVs, obtained after incubation with inflammatory cytokines (PIC) showed a time-dependent decrease in barrier function, not observed with EVs isolated in basal conditions (Figure 6). Furthermore, when cells were pretreated with EVs for 24 h and then wounded, autologous EVs-derived in non-inflammatory conditions showed to protect HCAEC from injury and participate in the re-establishment of a proper barrier function. Heterologous EV were shown to not influence the permeability of a HCAEC monolayer. Therefore, EVs do not seem to be angiostatic due to reduction in migration or increase in permeability of the HCAECs. However, we uncovered a potential beneficial role of the physiologic autologous EVs for recovery of injured endothelium and formation of a tight monolayer barrier.

Although EVs are believed to participate in endothelial inflammation, different reports suggest a protective role. Clinical studies showed indeed that EVs isolated from septic patients may also protect vascular tone and contractility and that, EVs-leukocytes conjugates correlate negatively with organ dysfunction and mortality (47, 48). Our data further suggest a critical role of EVs in barrier regulation and homeostasis. Indeed, while EVs produced under physiologic conditions may help recover the tight junctions between endothelial cells, EVs produced during inflammatory states may exert an effect on capillaries increasing permeability and favoring diapedesis and fluid leakage.

Extracellular vesicle cargo, to a certain extent, mirrors the miRNA-expressed repertoire of the parent cell and can cause biological changes to recipient cells. miRNAs are small non-coding RNA that play a role in regulating gene expression post-transcriptionally by repressing the expression of target mRNA. Several literature reports substantiate that EVs can deliver miRNAs to recipient cells, thus impacting the gene expression of the latter. Amongst these physiologic effects, it has been shown that miRNAs delivered by EVs can impair angiogenesis (49). Therefore, we interrogated the miRNA cargo of the autologous and heterologous EVs from control and PIC-stimulated cells. miRNA target genes included multiple genes known to mediate angiogenesis including those involved in classical VEGF signaling, cell cycle, and extracellular matrix. Extracellular vesicle miRNAs were found to target several functions relevant to endothelial cell function and angiogenic potential. Let-7 is integral to endothelial cell homeostasis (50). The let-7 family is known to have proangiogenic effects (51), but several instances of angiostatic activity have been reported. Some of the anti-angiogenic effects of let-7 are due to anti- proliferative effects. Since reduced proliferation was the most consistent effect of EVs both from control and pro-inflammatory cultures of ECs, we speculate that some of EV miRNA cargo, such as let-7, uniformly expressed by all EVs in this study, may have a key role via targeting multiple genes involved in proliferation (Table 1). Expression of let-7 has been shown to increase with age in cardiac stem-like cells (CSLC). Following treatment of CSLC with a let-7 mimic, their proliferation was significantly reduced (52). Angiogenesis is repressed by overexpression of let-7 and by silencing of the let-7 target gene HMGA2 in EC (53). miR-302 is a member of a cluster known to drive numerous functions critical to cell homeostasis and function including proliferation, differentiation, and pluripotency (54). MiR-302 has been shown to inhibit tumor angiogenesis by targeting VEGFA (55), and MACC1 (56). Circulating miR-302 has been identified as a potential biomarker for acute heart failure (57). The miR-23 cluster is highly expressed in EC and has been implicated in processes supporting angiogenesis (58–60). MiR-23 has also been implicated in atherogenesis, potentially by supporting a proinflammatory secretome in HCAEC (61) and has been linked to cardiomyopathy in mice (62). miR-126 has been implicated in the promotion of angiogenesis, however, numerous additional functions have also been observed. miR-126 was shown to downregulate NFκB-mediated PI3K/AKT/mTOR signaling (63) and PI3K inhibition has been implicated as a therapeutic target to inhibit tumor angiogenesis in multiple malignancies (64). Therefore, miR-126 delivered via EVs could exert a potential anti-angiogenic effect in the HCAEC via modulation of the latter pathway. miR-451, another miRNA detected in the EVs in this study has been found to be elevated in the blood of patients of coronary heart disease and inhibits proliferation in HUVECs (65). Inhibition of miR-451 in HUVECs has been demonstrated to promote tube formation and increase migration and invasion (66). miR-548 is downregulated in the pericoronary adipose tissue of coronary artery disease patients (67). miR-548 downregulated HMGB1 in human amniotic epithelial cells (68) and activation of HMGB1 in bone marrow stem cells stimulates proliferation and angiogenesis (69). Therefore, delivery of miR-548 to recipient HCAEC may engage the HMGB1 pathways and therefore reduce proliferation and tube formation, Our results also indicate that all of the miRNAs that were reported to induce angiogenesis in coronary endothelium post-ischemia (14) are, in fact, not detectable in our EV populations and perhaps it is not surprising that the functional miRNA cargo in our vesicles do not support pro-angiogenic mechanisms (Figure 7). Future studies are granted to validate the role of miRNA EVs in coronary angiogenesis and to identify potential therapeutic targets.

In this study, we showed the angiostatic and anti-proliferative effects of EVs from two EC sources in the absence and presence of inflammation and described their modulation of barrier function and recovery from wounding of human coronary endothelial cells. Our findings are important to consider when therapies with exogenous functionalized EVs are contemplated for enhancing post-ischemic coronary angiogenesis. Endogenous EVs from local or peripheral ECs may compete for uptake and counteract the therapeutic effects of the exogenous EVs. Therefore, short term treatment with small EV release inhibitors prior to exogenous EV delivery may maximize the potency of such therapeutic approaches.

## Data availability statement

The original contributions presented in this study are included in the article/Supplementary Material, further inquiries can be directed to the corresponding author/s.

## Author contributions

AD and RC conceived and designed the research. NC, AM, MB, and NM performed the experiments. NC, AM, MB, AD, and RC analyzed and interpreted the data. NC, AM, NM, RC, and AD drafted the manuscript. AD and JC edited and revised the manuscript. All authors approved the final version of manuscript.

## Conflict of interest

The authors declare that the research was conducted in the absence of any commercial or financial relationships that could be construed as a potential conflict of interest.

## Publisher’s note

All claims expressed in this article are solely those of the authors and do not necessarily represent those of their affiliated organizations, or those of the publisher, the editors and the reviewers. Any product that may be evaluated in this article, or claim that may be made by its manufacturer, is not guaranteed or endorsed by the publisher.

## Supplementary material

Supplementary Figure 1 HCAEC and HAMVEC EV C and EV PIC do not induce senescence in HCAEC. HCAEC incubated overnight with HCAEC and HAMVEC EC C and EV PIC were evaluated for senescence via detection of β-galactosidase. **(A)** HCAEC EV C and EV PIC did not induce senescence relative to untreated control, *p* = 0.0679. **(B)** HAMVEC EV C and EV PIC did not induce senescence relative to untreated control, *p* = 0.8179.
