# Supplementary figures and images for: Endothelial cell-derived extracellular vesicles impair the angiogenic response of coronary artery endothelial cells
Source: Front Cardiovasc Med. 2022 Jul 19;9:923081. doi: 10.3389/fcvm.2022.923081 (PMC9343725; doi:10.3389/fcvm.2022.923081)

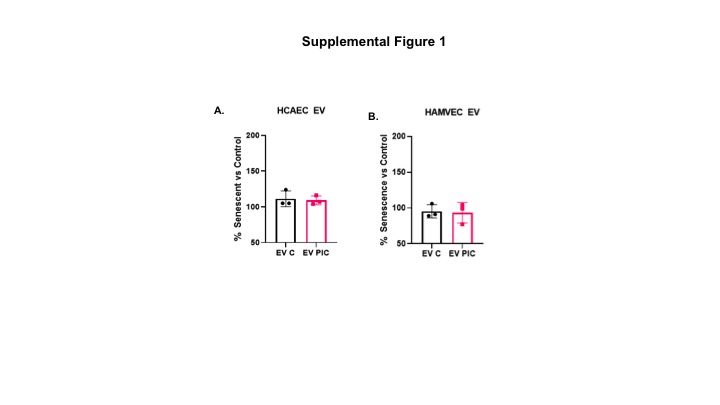

Supplement: Supplementary Figure 1 — HCAEC and HAMVEC EV C and EV PIC do not induce senescence in HCAEC. HCAEC incubated overnight with HCAEC and HAMVEC EC C and EV PIC were evaluated for senescence via detection of β-galactosidase. (A) HCAEC EV C and EV PIC did not induce senescence relative to untreated control, p = 0.0679. (B) HAMVEC EV C and EV PIC did not induce senescence relative to untreated control, p = 0.8179. [file Image_1.JPEG]
